# Brugada Syndrome Misdiagnosed As Acute Myocardial Infarction: A Case Report

**DOI:** 10.7759/cureus.26998

**Published:** 2022-07-18

**Authors:** Ahmad R. Khan, Salma Waqar, Amina Arif, Furqan Ul Haq, M. Isac Shah

**Affiliations:** 1 Internal Medicine, Hayatabad Medical Complex Peshawar, Peshawar, PAK

**Keywords:** coronary artery angiogram, exertional dyspnea, cardiac chest pain, acute st-elevation myocardial infarction, brugada ecg pattern

## Abstract

Brugada syndrome (BrS) is an autosomal-dominant condition mainly caused by defects in sodium channels causing ST-segment elevation in electrocardiograms (ECGs) in the V1 and V2 precordial leads, with ventricular tachyarrhythmias due to premature ventricular contractions, which increases the risk of sudden cardiac death. BrS usually presents in adulthood, with an average age of presentation of 41 years.

In this article, we describe a case of BrS diagnosed in a 36-year-old male having sudden cardiac arrest with no comorbidities such as hypertension, diabetes mellitus, smoking, or any valvular disease history. We then explain the ECG-based diagnosis, signs and symptoms, presentation at the emergency department, and treatment options.

## Introduction

Brugada syndrome (BrS) is a rare serious autosomal-dominant genetic arrhythmogenic disorder characterized by an electrocardiogram pattern of right bundle branch block (RBBB), ST-segment elevation (>2mm) in anteroseptal chest leads (V1-V3), and inverted T-waves in conjunction with an increased incidence of ventricular tachyarrhythmias and sudden cardiac death (SCD) in patients with a structurally normal heart. Approximately 20%-25% of patients with BrS have a genetic mutation in the SCN5A gene that causes a functional reduction in cardiac sodium channels resulting in a decreased influx of sodium into myocytes. BrS is diagnosed by characteristic electrocardiogram findings, significant events (e.g., syncope, palpitations, chest pain, nocturnal respiratory dyspnea, and family history of SCD below the age of 45), and other investigations (e.g., electrophysiology study, ajmaline provocation test, and genetic testing). Clinical presentation of BrS is related to recurrent life-threatening ventricular arrhythmia, self-terminating ventricular tachycardia, and documented idiopathic ventricular fibrillation increases the risk of SCD. The overall prevalence of BrS is estimated to be two to five in 1000 people, with a higher prevalence seen in Southeast Asia. It has a male preponderance, with a male-to-female ratio of 8-10:1. In most patients, symptoms manifest in the third or fourth decade of life, but the disease can occur at any age. The only effective and long-term treatment of BrS is the management of ventricular tachyarrhythmias with an automatic implantable cardioverter defibrillator (AICD). In an acute setting, the administration of appropriate antiarrhythmics (e.g., quinidine) and beta sympathomimetics (e.g., isoproterenol) is life-saving.

## Case presentation

We present herein the case of a 36-year-old male who presented to the Emergency Department (ED) with typical cardiac chest pain with substernal pressure, radiating to the left arm, nausea, tachycardia, acute anxiety, sweating, and later-developed syncope. The patient was thrombolysed with Streptokinase in the ED, as he was misdiagnosed with acute anterior wall myocardial infarction based on an initial electrocardiogram (ECG). Immediately after thrombolysis, the patient developed polymorphic ventricular tachycardia (VT), and CPR was started. He was successfully DC cardioverted with 13 shocks @ 270 J, and treatment started with vasopressors, Lidocaine, magnesium sulfate, and calcium gluconate. A 6Fr central venous sheath was passed into the internal jugular vein. 

**Figure 1:**
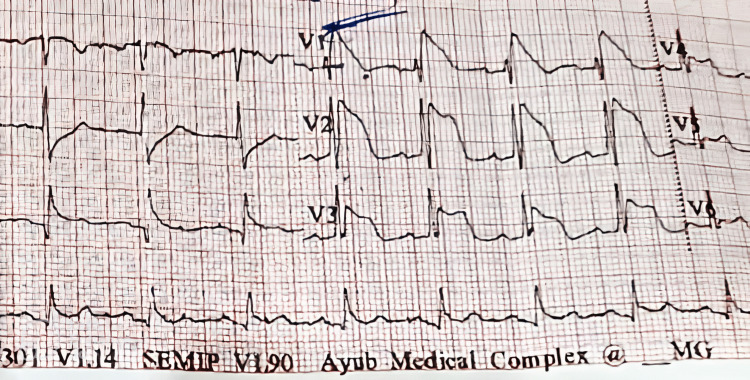
Coved ST-segment elevation in leads V1 and V2, followed by symmetric, negative T waves. V3 interestingly shows a partial saddle-back pattern, which is associated with Brugada syndrome.

After initial stabilization, the patient’s repeat ECG spontaneously showed coved ST elevation in leads V1 and V2 (Figure 1), with negative T waves typical of the type-1 Brugada pattern. The patient, subsequently, after stabilization, underwent coronary angiography to rule out coronary artery disease, which came back negative for ischemia, and his 2D ECHO was normal, with a normal ejection fraction of 58%.

There were no positive physical examination findings in either chest or cardiovascular exams, with no murmurs or extra heart sounds, a normal JVP, and no pedal edema. The patient was well-oriented in time, place, and person and maintained an oxygen saturation of 94% on room air with a normal BP of 129/91 and a pulse of 98. Lab results showed a normal creatinine of 0.7, magnesium of 2.5, negative Trop-I, and a potassium of 3.6.

According to the 2013 HRS/EHRA/APHRS criteria, the patient was definitively diagnosed with BrS as follows. The patient presented with sudden cardiac arrest and syncope resulting from ventricular tachyarrhythmia, which is the most significant clinical manifestation of BrS. He developed polymorphic ventricular tachycardia, which is more common in BrS patients who present with SCA or syncope. His ECG spontaneously showed a type-1 Brugada pattern with coved ST elevation in leads V1 and V2, followed by symmetric, negative T waves. The patient was a non-smoker, normotensive, and with no personal or family history of diabetes mellitus. His lipid profile showed normal lipid levels, with no family history of coronary artery disease. Subsequent coronary angiography was negative for Ischemia. However, the patient had a family history of sudden cardiac death.

## Discussion

BrS is a condition that causes ventricular tachyarrhythmia and is responsible for the occurrence of SCD in individuals with no structural heart deformities. Although it was considered a rare clinical entity when it was first introduced in 1992, it is now considered one of the most common causes of death among young individuals in certain countries [1]. Brugada et al. first described BrS in 1992. They described data from eight patients who presented with episodes of aborted sudden death, which occurred recurrently and were not explained by any known current diseases. Their shared similar clinical and electrocardiographic features compelled Brugada et al. to define them under the lines of a distinct syndrome that was quite different from idiopathic ventricular fibrillation [2]. Brugada and Brugada gave a typical ECG pattern of ST-segment elevation in leads V1 through V3 and incomplete RBBB and concluded that there was an increased risk of ventricular fibrillation resulting in SCD in these patients [2]. The characteristic ECG pattern in BrS usually manifests as a result of some precipitants in the form of drugs (e.g., sodium channel blockers, beta-blockers, TCAs, and alpha agonists) or metabolic abnormalities such as hypercalcemia, hyperkalemia, or hypokalemia. Intake of alcohol and cocaine can also precipitate these changes; otherwise, they usually remain concealed [1,2]. Brugada first described this condition as a functional cardiac disorder but later suggested that the underlying pathology may be either the refractory nature of cardiac muscles or extreme disturbances in the conductive properties of the ventricular muscles and conduction system of the Heart [2].

One of the strongest predictors of ventricular tachyarrhythmia in BrS is an abnormal baseline ECG that has a coved-type pattern with an ST-segment elevation that is > 0.2 mV in at least two contiguous precordial leads [3]. However, Veltmann et al. (2006) recognized in their prospective study that these typical ECG features tend to fluctuate. According to their study, 47% of patients with BrS did not present with a diagnostic baseline ECG Pattern. Out of 43 patients, only 1 presented with a continuously diagnostic coved-type ECG pattern that was considered characteristic of BrS in previous studies. They concluded that for correct risk stratification, repetitive ECG recording is crucial, as the prevalence of fluctuations between diagnostic and non-diagnostic ECGs in patients with BrS is very high [4].

Patients with ECG patterns characteristic of BrS have been identified as a distinct subgroup of male Thai patients who presented with ventricular fibrillation resulting in SCD [5]. A loss-of-function mutation in the SCN5A gene, encoding a subunit of sodium channels in cardiac tissue, accounts for 18-30% of BrS cases. The temperature-dependent changes in these sodium channels could explain the occurrence of a Brugada pattern in the ECG of febrile patients [6]. Smits et al.investigated the ECGs of a large number of patients with BrSand compared the basal ECG parameters in patients with and without the SCN51 gene mutation. PQ interval was the only parameter that was different between patients with and without this sodium channel mutation [7].

The management of BrS is either through pharmacological or non-pharmacological approaches. Pharmacological treatment includes intravenous isoproterenol/isoprenaline (enhancing the activity of ICa channels) with or without quinidine, which is a cardiac sodium channel blocker that stabilizes cardiac sodium channels to resolve the ST segment elevation [8,9].

Non-pharmacological approaches toward the acute arrhythmias or electrical storm in BrS include defibrillation and/or resuscitation if needed, and cessation of any arrhythmogenic drugs if suspected of having a role in the causation of the arrhythmias. ICD implantation in patients with BrS is reserved for those who present with aborted sudden death or severe symptoms such as seizure, syncope, or nocturnal dyspnea [10].

## Conclusions

The majority of BrS patients are diagnosed after SCD occurs in their family or after SCAs in people with low risk with no previous history of VT (i.e., people not protected by an ICD). ECG-based methods are best for diagnosing BrS at earlier stages in undiagnosed cases prior to the development of fatal arrhythmias. Genetic testing (SCN genes SCN5A and SCN10A, which encode subunits of cardiac sodium channels) of such families can also help in the prevention of BrS-induced ventricular arrhythmia by using prophylactic ICD placement.
